# Introductory Lectures

**Published:** 1850-04

**Authors:** 


### INTRODUCTORY LECTURES.

Those of our friends who have kindly sent us their Introductories, we trust will forgive us for having thus long left them unnoticed. We are not unmindful of their favors, nor ungrateful for the pleasure we have derived from reading them; but have been obliged to postpone them from unavoidable circumstances.

